# Smoking behavior and hypertension among health workers during the COVID-19 pandemic: a case study in Java and Bali-Indonesia

**DOI:** 10.3389/fcvm.2023.1146859

**Published:** 2023-08-14

**Authors:** Misnaniarti, Wahyu Pudji Nugraheni, Zainul Khaqiqi Nantabah, Fraschiska Rizky Restuningtyas, Risky Kusuma Hartono, Tety Rachmawati, Rofingatul Mubasyiroh, Asep Kusnali

**Affiliations:** ^1^ Public Health Faculty, Sriwijaya University, Kota Palembang, Indonesia; ^2^ Research Center for Public Health and Nutrition, National Research and Innovation Agency, Cibinong, Indonesia; ^3^ Health Development Policy Agency, Ministry of Health, Jakarta, Indonesia; ^4^ Department of Public Health, Universitas Indonesia Maju, Jakarta, Indonesia

**Keywords:** COVID-19, smoking, hypertension, health worker smoking, health workers, Java and Bali

## Abstract

**Background:**

Health workers who should be role models for the community not to smoke and live a healthy life are still consuming cigarettes. Java and Bali (especially Java) are the biggest contributors to health worker deaths due to COVID-19 in Indonesia. This study aims to determine the association of smoking behavior and hypertension among health workers in Java and Bali. The researchers conducted this study in 2021 and designed it with a cross-sectional design. Data was collected online using the *Lime Survey* as a data collection tool. The data analysis used was logistic regression to determine the association of smoking and hypertension.

**Result:**

A number of 7.6% of health workers were still smoking and 10.4% were suffering from hypertension during the COVID-19 pandemic. This study shows that the proportion of health workers with hypertension is two times that of smokers (18.0%) compared to non-smokers (9.8%). Logistic regression showed that smoking has a 20% higher risk of developing hypertension (OR = 1.97; 95%CI = 1.01–1.41; *p* = 0.034).

**Conclusion:**

Among health workers on the islands of Java and Bali, there are still many who smoke, and this puts them at a higher risk of experiencing hypertension.

## 1. Introduction

Tobacco use has caused the death of more than 200 million people over the last 30 years (1). Cigarette smoking and other tobacco use impose a growing public health burden globally (2). More than 8 million people die yearly from smoking tobacco. Approximately 5 million people are killed annually by tobacco use (3). The harmful effects of smoking are responsible for illness, death, and impoverishment, extending well beyond individual and population health as billions of dollars in lost productivity and healthcare expenditure (4).

Indonesia has the third-highest smoking prevalence globally, only after China and India (5). According to the World Bank report, nearly 40% of the total population with age equal to or above 15 years smoke, while 76% of Indonesian males are the highest smoker group globally (6). This nation contributed to 60 million active smokers in the world. The smoking prevalence has remained high from 34.7% in 2007, 36.5% in 2013, and 33.8% in 2018 (6, 7). Based on the WHO Framework Convention on Tobacco Control, health workers have an important role to play in reducing the prevalence of smokers through smoking cessation efforts (8), including in Indonesia.

Indonesia has health workers spread across Indonesia to help smokers stop smoking. Smokers are also more likely to quit smoking when they receive counseling from health workers (9). Unfortunately, there are still health workers who are active smokers, so this condition contributes to the prevalence rate of smokers and hinders smoking cessation programs in Indonesia (10, 11). Health workers who should be role models for the community not to smoke and live a healthy life are still consuming cigarettes (12, 13). Health workers should not smoke cigarettes because they should become role models for the community to control tobacco.

East Java and Central Java are the largest tobacco industrial areas in Indonesia (14). Most governments on the islands of Java and Bali have implemented Smoke-Free Areas. Regional rules regarding smoke-free areas have been introduced at the provincial and district/city levels. These regulations encompass health care facilities as one of the designated smoke-free areas (15–17). However, there are still many smoking health workers in the area. Some health workers on the island of Java smoke because they are tired after work (11). Study in Grobogan, Central Java found that 35% of health service employee were smoked (18), likewise it was found that 36% of health workers aged 31–40 in Bali (10). The negative impact of smoking behavior also threatens the health condition of health workers. Study said that physician smokers tend to be associated with hypertension and obesity (19). The condition of hypertension in health workers in Indonesia can be seen in research conducted in Palembang (20).

Smoking health workers exacerbates the pandemic because smoking is a risk factor for the severity and death of Covid-19 (21). Furthermore, another study also explained that the prediction of the incidence of hypertension in workers by 9% could occur due to the influence of daily cigarette consumption, and the number of cigarettes used reached 10–20 cigarettes/day (22). However, the impact of smoking behavior on health workers still requires further evidence, especially in Java and Bali, because they have the most significant number of health workers in Indonesia. Even so, there is little evidence of smoking behavior among health workers and its implications for smoking-related diseases in Java and Bali, Indonesia. This study aims to determine the association of smoking behavior and hypertension among health workers in Java and Bali. This study is necessary to provide evidence of smoking prevalence from the health personnel sector to strengthen the tobacco control effort in Indonesia.

## 2. Material and methods

This cross-sectional study used data from a study mapping the risk behavior of non-communicable diseases among employees at the relevant Provincial/District/City/ Regional Technical Implementation Units (UPTD) Health Offices in Java and Bali. The study population was health workers in all areas of Java and Bali and all relevant provincial/city/district/UPTD health office employees. The majority of health workers who died due to COVID-19 came from the medical profession, namely 730 people, 670 nurses, and 388 midwives. East Java occupies the top position as the province with the highest number of deaths from health workers in Indonesia, namely 643 people. West Java and Central Java are followed with a total of 225 health personnel deaths and 193 people, respectively. Health workers in Indonesia who died due to COVID-19 and health workers who died due to comorbid or congenital diseases. Java and Bali (especially Java) are the biggest contributors to the deaths of health workers in Indonesia (23).

This research uses online data collection because the ongoing threat of the COVID-19 outbreak still needs to be monitored. There is an appeal from the Indonesian government to continue limiting people's movements due to COVID-19. Researchers conducted pre-test trials to determine the validity and reliability of the questionnaire and estimate the time required for respondents to complete it. This trial was conducted online in August 2021 in West Nusa Tenggara Province. At the trial stage, the validity of the smoking question was good, but the reliability was low. To increase reliability, the research team improved the narrative of the smoking question so that it was easier for respondents to understand and be consistent in answering, then the questionnaire being used for collecting data. The trial showed a self-report duration of 10–15 min. This study was conducted from September to October 2021. Questionnaire have been changed to digital form in the LimeSurvey application. LimeSurvey is an online survey platform that has easy facilities for preparing templates, sending surveys, and analyzing results at affordable prices. The questionnaire link are distributed to all respondents through the provincial health office structure to the puskesmas or other UPTD Health Office. The UPTD of the health office includes regional hospitals, health laboratories, and health centers. The sampling technique for online data collection can be non-probability sampling. This method involves selecting a sample of participants based on non-random criteria. Non-probability sampling may be less representative than probability sampling, but it can still provide useful information (24).The research received significant respondents (21,095) at the end of data collection. Using the formula of the Lemeshow health study sample for two-sample hypothesis testing of proportions (25), an estimated prevalence of hypertension in the smoker group was 21,22%, and the prevalence of hypertension in the non-smoker group was 19,06% based on the previous study (26) and a 95% confidence interval 0.10 wide (0.05 on either side), the minimum sample required was 16,978. Thus, the number of samples we analysed was larger than the minimum needed.

The outcome variable in this study was hypertension from self-reporting blood pressure in the last month. Hypertension was categorized inti two groups: normal if systolic <140 mmHg and diastolic <90 mmHg; hypertension if systolic ≥140 mmHg or the diastolic ≥90 (27).

The independent variable was smoking behavior and socio-demographic factors, including education, age, and gender. The researchers divided respondents who smoke into two groups: respondents who do not smoke (never smoke or former) and respondents who smoke (occasional and every day).

Data were analyzed using SPSS software. The data analysis used is a descriptive statistical analysis to see the characteristics of the data and logistic regression analysis to determine the association between smoking behavior and the incidence of hypertension. The researchers measured the significant relationship at the 0.05 significance level.

## 2. Results

### 2.1. Characteristics of respondents

Table 1 is a descriptive demographic of the character of the Provincial/City/Regency/UPTD Health Office employees who participated in this survey. The results show the majority who took part in this survey were female, amounting to 78.8% (16,630), while only 21.2% (4,465) were male. Respondents with education level Diploma 1–3 are the most respondents with a percentage of 51.5% (10,867). The second-largest number of respondents, when viewed from the level of education, are respondents with Diploma to bachelor education, which is 37.2% (7,853). Also, in Table 1, respondents aged 30–34 years are respondents with the highest proportion, namely 18.2% (3,843). The second-largest age group is respondents aged 35–39, as much as 17.1% (3,602). Several 7.6% of health workers were still smoking and 10.4% were suffering from hypertension during the COVID-19 pandemic.

**Table 1:** Characteristics of health workers in Java and Bali in 2021.

Characteristics of Respondents	Total (*n*)	Percentage (%)
Gender		
–Male	4,465	21.2
–Female	16,630	78.8
Age		
–18–24	665	3.2
–25–29	3,423	16.2
–30–34	3,843	18.2
–35–39	3,602	17.1
–40–44	3,254	15.4
–45–49	2,633	12.5
–50–54	2,397	11.4
–55–59	1,254	5.9
–≥60	24	0.1
Education		
–Junior High School	97	0.5
–Senior High School	1,455	6.9
–Diploma 1–3	10,867	51.5
–Diploma4/bachelor	7,853	37.2
–Postgraduated/doctoral	823	3.9
Smoking behavior		
–No	19,485	92.4
–Yes	1,610	7.6
Hypertension		
–Normal	18,903	89.6
–Hypertension	2,192	10.4
Total	21,095	100.0

### 2.2. Smoking behavior of health workers in Java and Bali

Table 2 describes the two groups of smokers: researchers divided respondents who smoke into two groups: respondents who do not smoke every day (sometimes) and respondents who smoke every day. Among them, 34 respondents (7.0%) who did not smoke daily reported spending more than 25 cigarettes weekly. The highest number of smokers consume 1–12 cigarettes weekly (381 respondents or 78.7%). In addition, the average number of cigarettes consumed daily is 1–12 cigarettes a day (80.5%), but some employees smoke more than 25 daily (1.9%). Most smokers smoked outdoors (1,548 respondents or 96.1%). However, some smokers smoke indoors (62 respondents or 3.9%).

**Table 2:** Overview of cigarette consumption of health workers in Java and Bali in 2021.

Smoking Behavior	Total (*n*)	Percentage (%)
The average number of cigarettes in a week		
–1–12 sticks	381	78.7
–13–24 sticks	69	14.3
–≥25 sticks	34	7.0
The average number of cigarettes per day		
–1–12 sticks	906	80.5
–13–24 sticks	199	17.7
–≥25 sticks	21	1.9
Smoking locations		
–Indoor	62	3.9
–Outdoor	1,548	96.1

### 2.3. Association between smoking behavior and hypertension category

Table 3 shows that the proportion of health workers with hypertension is two times that of smokers (18.0%) compared to non-smokers (9.8%). A significant association between smoking behavior and the hypertension category of health workers in Java and Bali in 2021. That can be seen from logistic regression showed that smoking has a 20% higher risk of developing hypertension (OR = 1.97; 95%CI = 1.01–1.41; *p* = 0.034).

**Table 3:** Logistic regression between smoking behavior and hypertension of health workers in Java and Bali in 2021.

Smoking behavior	Hypertension		* *	* *
	No	Yes	*OR (95% CI)*	*p-value*
	*N* (%)	*N* (%)		
Normal	17,582 (90.2)	1,903 (9.8)	1	
Hypertension	1,321 (82.0)	289 (18.0)	1.97 (1.01–1.41)	0.034

## 3. Discussion

This study's main finding proves that health personnel still become active smokers, 8.38% of the total respondents. Nevertheless, health personnel should give an example to stop smoking. It becomes essential to do a depth analysis. The findings of this study are still higher than the study in the U.S., which found that the prevalence of smoking among health professionals was minor than 6% (28). This study's finding is also higher than Malaysia's, which found that the overall smoking prevalence of health workers was 1.6% (29).

Currently, the prevalence of tobacco use in healthcare workers worldwide is unknown (30). Research showed that compared to men in the general population in low- and lower-middle-income countries, including Indonesia, male health workers have a 20% higher prevalence of smoking among health workers. However, it is still lower than in Armenia, Syria (30%), Pakistan, and Tunisia (50%). Meanwhile, the prevalence of smoking among female health workers in Indonesia, Nepal, and Syria tends to have a lower prevalence (<5%) and is highest in Armenia and Pakistan (<15%) (30). These results align with research in Asia which found that the smoking behavior of male health workers is higher than that of female health workers (31).

Previous research on Sumatra, Indonesia, found that 75.6% of Tebing Tinggi City health service employees smoked (32). Several factors, including knowledge about smoking and the smoking behavior of colleagues, can influence smoking behavior among health workers. The behavior of co-workers is very influential; in fact, up to 32% of smoker respondents share cigarettes with co-workers, and 54% do not recommend that co-workers who smoke move to the designated smoking room available (18). As many as 17.8% of health service employees and smokers who do not agree with the implementation of smoke-free areas, but they agree that smoking also impacts non-smokers around them (32).

On the contrary, a study in Italy found that smoking prevalence among hospital employees was 47% (42% among doctors and 43% among nurses); 30% admit to smoking in hospitals, and three-quarters of smokers want to quit (33). This figure is greater than the findings of this study. The study implies that smoking among healthcare professionals may still be very high and maybe twice the level observed in the general population included in this study. It could happen because health workers from high-income countries tend to have lower prevalence rates than those from middle- and low-income countries (30, 34).

Cases of health workers who smoke mostly come from those with low incomes, fewer years of formal education, and specific nursing specialties (25). Another study showed that work stress was most associated with nurses who graduated from high school, lack of nurses, nurses who had many overtime hours, nurses with the most shifts, and the lowest level of education (35). The findings are similar to this study. Another factor is the cultural norms of smoking behavior which are still readily accepted by the Indonesian population so that health personnel can still reach these norms, so they decide to become active smokers (36).

This study also proves that health personnel who smoke have a history of grade 1 hypertension (14.7%), grade 2 hypertension (2.9%), and critical hypertension (0.4%). Various studies in Indonesia are also in line with the result of this study. They revealed the negative impacts of smoking from decreased productivity experienced by young people, which can cause catastrophic diseases, including diabetes, heart disease, and various types of cancer (1, 37, 38). In addition to causing an addictive effect, this nicotine product can cause hypertension by binding to the blood. An unhealthy lifestyle, such as consuming junk food and lacking physical activity, also supports this, but it was unobserved in this study.

Hypertension is a global health problem, especially in low- and middle-income countries (39). Based on a study by Prakash Ghimire et al., the prevalence of hypertension among health workers in Nepal was 35.31%. The factors influencing this are age, education level, marital status, low vegetable and fruit consumption, low physical activity, alcohol consumption, and stress levels (40). Research from Po-Ya Chang on hypertension in nurses in Taiwan informs us that factors that influence nurses who suffer from hypertension are stress levels, age, BMI, working hours a week, and taking care of family members (41).

The results of the research conducted by Tantya Issumantri stated that smoking employees at the Kulon Progo puskesmas continued to smoke as usual in the puskesmas area, even though there had been regulations prohibiting smoking in the puskesmas environment. Even the officers did not reprimand or remind them if visitors to the puskesmas smoked in that environment (42). That shows that health workers are also less concerned about smoking behavior, which can impact health. Alya Binda Ulinuha et al. corroborated this research, concluding that health workers who smoke are already aware of the adverse effects of smoking. However, they believe smoking will bring them peace, health, and the absence of disease symptoms. Health workers who smoke intend to quit, but determination and execution are still lacking (11).

Another study finding is that the percentage of health workers exposed to cigarette smoke daily is still relatively high at 26.36%. It has the same finding with the exposure to second-hand smoke of health workers in Malaysia; it was 21% and 39% at home and in public places (29). It happens because certain spots, such as parking lots, behind the building, or outside areas close to the building's surroundings, do not cover the smoke-free area. Another extreme possibility is exposure to second-hand smoke in inpatient, office management, and administration rooms (33, 34). In addition, 85% of the population is still exposed to cigarette smoke in public places, supported by the weak regulation of smoke-free areas in public transportation, universities, and education places in Indonesia compared to other Asian countries (43). Air quality is also affected by cigarette smoke. Air measurements conducted at several primary healthcare facilities in Spain that are part of the Smoke-free Primary Health Care Programme have shown that there is still exposure to cigarette smoke in specific areas such as staff rooms and reception areas. These areas had the highest levels of airborne nicotine with maximum values of 1.40 and 0.60 mg/m3, respectively (19). This second-hand smoke issue causes a somewhat dangerous impact on clinical and hospital health services.

Health personnel should not smoke cigarettes because they should become a role model for the community to control tobacco. The 2024 National Medium-Term Development Plan targets 350 districts/cities with 40% of primary health care providing Stop Smoking Services by 2024 in Indonesia (44). Fulfilling this target may experience obstacles whether health personnel is still against tobacco control efforts. In addition, Indonesia's National Health Insurance (NHI) had to cover IDR 10.5–15.5 trillion or around 56%–59% of the total healthcare costs for smoking-related diseases in 2019 (45). Those diseases are suffered by NHI participants which are include health and non-health personnel. Data regarding the burden of claims for smoking-related illnesses suffered by health personnel nationally still requires further research. This burden requires the role of health workers and awareness of health workers not to become smokers.

The COVID-19 pandemic has put health systems around the world under stress. During the handling of Covid-19, health workers were the party most at risk of exposure to hazards, including exposure, long working hours, psychological stress, fatigue, work fatigue, stigma, and physical (46). The health workforce is one of the six building blocks of the health system recognized by WHO (47), which is the key to the health system, having the ability to deal with external shocks such as outbreaks (48).

The ongoing role of health workers in long-term recovery after a pandemic provides evidence for the role of health workers in pandemic preparedness and strengthening the public health system (49). The vital role of health workers in dealing with a pandemic includes (1) facilitating vaccinations and appointments in all areas with low vaccination rates. Health workers deal with various challenges in the pandemic, including virus problems, mental health issues, and obstacles in vaccine access. They tackle these challenges by (1) building trust with the community through involvement in community activities, increasing community recognition, and promoting accessibility to support services; and (2) sustaining progress in public health despite funding instability (50). The role of health workers is not only as assistants but also as agents of social change and community activation (51). This critical role leaves Health workers well-positioned to respond in times of crisis but also to work for long-term recovery and rebuilding, promoting community resilience (52).

Indonesia already has the principal tobacco control regulations that apply to all circles, namely Government Regulation no. 109 of 2012 concerning the Safety of Materials Containing Addictive Substances in the Form of Tobacco Products for Health (53). However, the regulation, which is already ten years old, urgently needs revision to keep up with current developments. One of these developments is the need to include health workers in tobacco control efforts. Minister of Home Affairs Regulation Number 17 of 2021 concerns guidelines for preparing local government work plans for 2022. This regulation will at least increase the number of Public Health Centers (*Puskesmas*) that support tobacco control and establish smoking cessation services. On the other hand, Indonesia still needs to sign or ratify the FCTC; MPOWER guides the country's national health policy and tobacco control strategy. However, in collaboration with non-governmental organizations (NGOs), the government has demonstrated some commitment to addressing tobacco-related health issues (14).

This study is one of the research projects that reveal a controversial issue, and they are the findings of smoking behavior among civil servants and health personnel. To the author's knowledge, there have been rare instances of this conducted in Indonesia. However, conducting an online study raises the potential for bias and uneven distribution of the sample, including more women in the sex group, which is a limitation of this study. Future studies can improve the result of this study by conducting face-to-face in-depth interviews with health workers to explore the causes of their continuing smoking behavior and the most appropriate cessation method for them.

## 4. Conclusions

This study shows that there are still smokers among health workers during the COVID-19 pandemic (7.6%), as well as those who suffer from hypertension (10.4%). The proportion of hypertension is higher in smokers (18.0%). and smokers have a higher risk of developing hypertension. The Government of Indonesia and medical and health associations need to formulate strict policies and sanctions for the health personnel proven to have smoking behavior. Health facilities need to apply strict sanctions to health personnel proven to smoke. All health personnel must be role models for not smoking and support Indonesia's goal of developing a smoking cessation clinic in primary health care. Researchers must conduct further studies to investigate the causes of smoking behavior among health workers and identify the most suitable cessation methods to encourage them to quit smoking.

## Data Availability

The data that support the findings of this study are available from the Data Management Laboratory of the National Institute of Health Research and Development (NIHRD), Ministry of Health of Indonesia. Data can be made available after approval of a written request to the Data Management Laboratory—NIHRD. Requests to access the datasets should be directed to tetyr272002@gmail.com.
